# Enabling Molecular-Level Computational Description of Redox and Proton-Coupled Electron Transfer Reactions of Samarium Diiodide

**DOI:** 10.1021/acs.jpca.3c00418

**Published:** 2023-04-19

**Authors:** Jonas Himmelstrup, Vidar R. Jensen

**Affiliations:** Department of Chemistry, University of Bergen, Allégaten 41, N-5007 Bergen, Norway

## Abstract

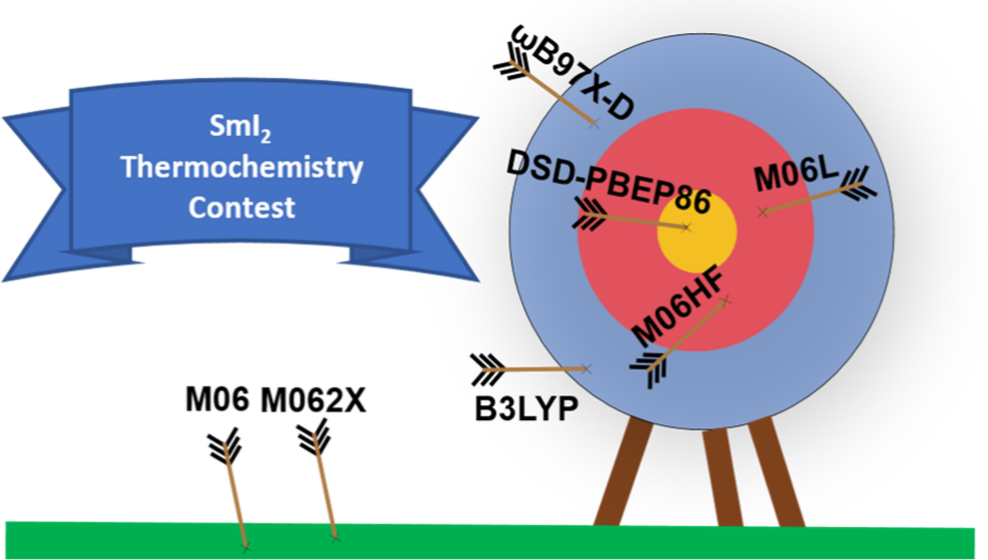

Samarium diiodide (SmI_2_, Kagan’s reagent) is a one-electron reductant with applications ranging from organic synthesis to nitrogen fixation. Highly inaccurate relative energies of redox and proton-coupled electron transfer (PCET) reactions of Kagan’s reagent are predicted by pure and hybrid density functional approximations (DFAs) when only scalar relativistic effects are accounted for. Calculations including spin–orbit coupling (SOC) show that the SOC-induced differential stabilization of the Sm(III) versus the Sm(II) ground state is little affected by ligands and solvent, and a standard SOC correction derived from atomic energy levels is thus included in the reported relative energies. With this correction, selected meta-GGA and hybrid meta-GGA functionals predict Sm(III)/Sm(II) reduction free energies to within 5 kcal/mol of the experiment. Considerable discrepancies remain, however, in particular for the PCET-relevant O–H bond dissociation free energies, for which no regular DFA is within 10 kcal/mol of the experiment or CCSD(T). The main cause behind these discrepancies is the delocalization error, which leads to excess ligand-to-metal electron donation and destabilizes Sm(III) versus Sm(II). Fortunately, static correlation is unimportant for the present systems, and the error may be reduced by including information from virtual orbitals via perturbation theory. Contemporary, parametrized double-hybrid methods offer promise as companions to experimental campaigns in the further development of the chemistry of Kagan’s reagent.

## Introduction

Samarium diiodide, also known as Kagan’s reagent, is an important reductant and, when combined with a Brønsted acid, a proton-coupled electron transfer (PCET) agent in a multitude of organic reactions.^1,2^ Its PCET properties have also led to a recent breakthrough in low-temperature, homogeneously catalyzed ammonia synthesis, in which water, for the first time, acts as a proton source and stunning nitrogenase-like catalytic activities are obtained.^3^ The widespread and varied use of Kagan’s reagent in organic chemistry and its new role in the development of alternative ammonia production processes should be complemented by molecular-level computational studies to provide insights and to spur further progress.

Illustrating the drive to obtain fundamental insights via synergistic use of calculations and experiments are a series of recent studies of the solution-based coordination chemistry of SmI_2_^2,5−7^ and of C–C bond formation reactions initiated by a single-electron transfer (SET) from Sm(II) to redox-active ligands.^8^ However, only a couple of computational studies have so far targeted the energetics associated with the one-electron oxidation of SmI_2_.^5,9^ The reason for this lack of computational studies of the very process that is key to the reactivity of SmI_2_ is presumably rooted in the challenges involved in describing this one-electron oxidation using standard density functional approximations (DFA). These challenges have been thoroughly demonstrated by Maron and Perrin and co-workers,^100^ who concluded that it is “more imperative than ever” to identify “a general method or strategy that will be simple and effective” in describing the redox chemistry and SET processes of SmI_2_.

Here, we answer this call by analyzing two exemplary processes key to the reactivity of SmI_2_: the one-electron reduction of SmI_2_(THF)_5_^+^ and the PCET-relevant bond dissociation free energy (BDFE) of an O–H bond of SmI_2_(THF)_4_H_2_O, both in tetrahydrofuran (THF) solution. The performance of density functionals of up to the fifth rung of Jacob’s ladder^10^ is compared to experimental estimates. To further validate the PCET-relevant performance, BDFEs for a smaller model complex, SmI_2_(H_2_O)_5_, are compared to those of the coupled cluster method CCSD(T). Finally, the results are rationalized in terms of electron correlation, exchange, and the delocalization error (DE) of DFAs. The associated insights and recommendations will be useful for future computational contributions to the understanding and development of the rich chemistry driven by electrons from SmI_2_.

## Computational Methods

All density functional theory (DFT) calculations were performed using the C.01 version of the Gaussian16 package.^11^ For spin multiplicities >1, unrestricted calculations were performed. Significant spin contamination was not observed.

### Geometry Optimizations

The hybrid meta exchange–correlation functional PW6B95^12^ complemented by Grimme-type D3 empirical dispersion^13^ with Becke–Johnson damping,^14,15^ here labeled PW6B95-D3(BJ), was used for geometry optimizations. The PW6B95-D3(BJ)-optimized geometry of SmI_2_(THF)_5_^+^ compares excellently with the corresponding single-crystal X-ray structure, with the optimized Sm–O and Sm–I distances only being slightly (ca. 1%) longer than those of the X-ray structure (see Section S2.1 in the Supporting Information). A similar pattern of slight overestimation of Sm–ligand distances is seen when comparing the optimized Sm–O and Sm–I distances of SmI_2_(THF)_4_H_2_O with those obtained from in situ X-ray absorption spectroscopy on a 1:1 mixture of SmI_2_ and water in THF.^16^ More generally, the PW6B95 functional, with or without empirical dispersion corrections, has been found to perform well across a broad range of transition-metal chemistry,^17^ including the prediction of spin-state stability,^18^ as well as main-group chemistry.^19,20^

The input geometries of SmI_2_(THF)_5_/SmI_2_(THF)_5_^+^ were taken from ref (9), in which the structure of SmI_2_ in THF has been explored computationally. In the latter work, SmI_2_ was found to coordinate five THF molecules, forming the coordinatively saturated SmI_2_(THF)_5_, with the two iodide ligands located trans to each other. This is consistent with the single-crystal structure determined for SmI_2_ crystallized from THF.^21^ For SmI_2_(THF)_4_H_2_O and SmI_2_(THF)_4_OH, input geometries were generated by replacing, using a molecular builder, a THF moiety by water and hydroxide, respectively. Similar procedures were followed when generating input geometries for  and .

Solvent effects were included in the geometry optimizations by using the SMD continuum solvation model for THF.^22^ The cavity was constructed by adding the solvent radius to the unscaled atomic radii (surface = SAS). The self-consistent field (SCF) convergence criterion was tightened 10-fold (SCF (conver = 9)) compared to the default, and the wavefunction was tested for instabilities (stable = opt) at the start of the geometry optimization. Geometries were optimized to a maximum force of 1.5 × 10^–5^ au (opt = tight). For SCF energies and gradients, numerical integrations were performed using Gaussian’s “ultrafine” grid (a pruned 99,590 grid with 99 radial shells and 590 angular points per shell), whereas Gaussian’s “finegrid” (a pruned 75,302 grid with 75 radial shells and 302 angular points per shell) was used for the CPHF analytical Hessian calculations. The eigenvalues of the analytically calculated Hessian were used to characterize stationary points, confirming positive curvatures (or no imaginary frequencies) for the minima.

The basis sets for geometry optimization were as follows: for H and C, correlation-consistent valence double-ζ plus polarization (cc-pVDZ) basis sets were used.^23^ For O, a correlation-consistent valence double-ζ plus polarization basis set augmented by diffuse s, p, and d functions (aug-cc-pVDZ) was used.^23−27^ For I, a relativistic 28-electron Stuttgart/Cologne effective core potential (ECP28MDF) was used in conjunction with an accompanying correlation-consistent valence double-ζ plus polarization basis set augmented by diffuse s, p, and d functions (aug-cc-pVDZ-PP).^28,29^ For Sm in oxidation state +II, a quasi-relativistic 52-electron Stuttgart/Cologne ECP (ECP52MWB) was combined with an accompanying (7s6p5d) primitive basis set contracted to [5s4p3d].^30,31^ With the ECP incorporating the unpaired electrons, singlet spin multiplicity was used when geometry optimizing spin-septet Sm(II) complexes. Similarly, for Sm in oxidation state +III, a quasi-relativistic 51-electron Stuttgart/Cologne ECP (ECP51MWB) was combined with an accompanying (7s6p5d)/[5s4p3d] basis set. With the ECP incorporating the unpaired electrons, singlet spin multiplicity was used when geometry optimizing spin-sextet Sm(III) complexes.

The ECPs and basis sets of the geometry optimizations were taken from the basis set exchange server,^32−34^ except for Sm, for which the Stuttgart/Cologne website was used.^35^ See Table S1 for an overview of the basis sets used for geometry optimization.

### Single-Point Calculations

Single-point (SP) calculations at the optimized geometries were performed using more flexible basis sets and a range of different computational methods (see Table S2 for an overview). The SCF density-based convergence criterion was set to rms <1.0 × 10^–5^ and maximum change <1.0 × 10^–3^ (keyword SCF (conver = 5)) for the DFT calculations. For the double-hybrid and coupled-cluster calculations, the SCF convergence criterion was set to “tight”, and the chemical valence electrons of O, C, and H atoms were included in the correlation treatment, whereas the core electrons were frozen. For I, the 4d electrons were included in the correlation treatment in addition to the 5s and 5p valence electrons. For Sm, the 4d, 5s, 5p, 4f, and 6s electrons were included in the correlation treatment.

Unless otherwise stated, solvent effects of THF were included in the SP calculations via the SMD continuum solvation model,^22^ with the cavity built using scaled atomic radii. Natural atomic charges and electron populations were obtained from natural bond orbital (NBO) analyses using the NBO7 software.^36^ In the double-hybrid calculations, the option “density = current” was included to ensure that the NBO analyses were performed on the final double-hybrid density.

The basis sets used in the SP calculations were as follows: for H and C, correlation-consistent valence triple-ζ plus polarization (cc-pVTZ) basis sets were used.^23^ For O, a correlation-consistent valence triple-ζ plus polarization basis set augmented by diffused s, p, and d functions (aug-cc-pVTZ) was used.^23−27^ For I, a relativistic Stuttgart/Cologne 28-electron ECP (ECP28MDF) was used in conjunction with an accompanying valence triple-ζ plus polarization basis set augmented by diffuse s, p, and d functions (aug-cc-pVTZ-PP).^28,29^

For Sm, a quasi-relativistic 28-electron Stuttgart/Cologne ECP (ECP28MWB) was used in conjunction with an accompanying segmented (14s13p10d8f6g)/[10s8p5d4f3g] basis set.^37,38^ With no unpaired electrons included in the ECP, septet and sextet spin multiplicities were used in the SP calculations on Sm(II) and Sm(III) complexes, respectively.

The ECPs and basis sets of the SP calculations were taken from the basis set exchange server,^32−34^ except for Sm, for which the Stuttgart/Cologne website was used.^35^ See Table S3 for an overview of the basis sets used in the SP calculations.

Basis set superposition errors (BSSE) affecting the BDFE of SmI_2_(THF)_4_H_2_O (fragments: SmI_2_(THF)_4_OH and H) and SmI_2_(H_2_O)_5_ (fragments: SmI_2_(H_2_O)_4_OH and H) were calculated using the counterpoise method in conjunction with the above SP basis sets for DFT. The functionals selected for BSSE calculation were M06L-D3, PW6B95-D3(BJ), and DSD-PBEP86, thus spanning rungs 3–5. The BSSE was found to be small in all cases (0.1–0.3 kcal/mol for the regular DFAs and 0.7 kcal/mol for the double-hybrid method; see Tables S7 and S8), and the reported BDFEs are thus not corrected for BSSE.

Estimates of spin–orbit coupling (SOC) effects were obtained via SP calculations using the 5.03 version of the ORCA package.^39^ Using the second-order Douglas–Kroll–Hess (DKH) Hamiltonian^40,41^ and the SMD solvation model throughout,^22^ all-electron, complete active space SCF (CASSCF) calculations with an active space consisting of seven orbitals and six and five electrons, respectively, for Sm(II) (CASSCF(6,7)) and Sm(III) (CASSCF(5,7)) were used to generate zeroth-order wavefunctions for the subsequent strongly contracted N-electron valence perturbation theory (SC-NEVPT2) treatment,^42−44^ in which the correlation treatment involved the same electrons as described above for the coupled-cluster calculations. The CASSCF wavefunctions were averaged over the seven (^7^F) and 21 (^6^H^o^) roots of the spin-septet and sextet atomic Sm^2+^ and Sm^3+^ terms, respectively. The converged active orbitals were quite pure Sm 4f orbitals. SOC effects were estimated via quasi-degenerate perturbation theory (QDPT)^45,46^ using an approximate Breit–Pauli mean-field SOC operator, termed RI-SOMF(1X),^47^ in which the exchange term is obtained via one-center exact integrals including the spin-other orbit interaction, and the Coulomb term is computed using the resolution of the identity approximation. The QDPT treatment used a basis of roots of the Born–Oppenheimer Hamiltonian, with the matrix elements computed over the state-averaged CASSCF wavefunctions, but with NEVPT2 energies along the diagonal of the QDPT matrix, accounting for dynamic correlation effects.

The orbital basis sets used in the all-electron SOC calculations were built-in ORCA basis sets, as detailed in the following: O, C, and H atoms were described by DKH-def2-TZVP basis sets, which are def2-TZVP basis sets^48^ recontracted for DKH by D. A. Pantazis. I atoms were described by segmented all-electron relativistically contracted (SARC) basis sets of the SARC-DKH-TZVP type.^49^ Sm atoms were described by the SARC2-DKH-QZVP basis set.^50^ Accompanying auxiliary SARC/J basis sets were used for Coulomb fitting, implying decontracted def2/J sets for O, C, and H atoms,^51^ and SARC/J sets for I,^49^ and Sm.^52^

### Calculation of Gibbs Free Energies

Thermochemical corrections (*G*_PW6B95-D3(BJ) qh_^THF 298K^) to give Gibbs free energies were calculated at the geometry optimization level at 298 K using the ideal gas, rigid rotor, and harmonic oscillator approximations, except that frequencies below 100 cm^–1^ were shifted to 100 cm^–1^ when calculating the vibrational entropy (i.e., the quasi-harmonic oscillator approximation, here indicated using a subscript “qh”)^53^ to correct for the breakdown of the harmonic oscillator model for entropies of low-frequency vibrational modes. The Gibbs free energy of a system was thus obtained using eq 1.
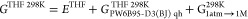
1

 is the standard state correction corresponding to a 1 M solution, amounting to 1.89 kcal/mol (=*RT* ln(24.46)) at room temperature. *E*^THF^ is the potential energy resulting from an SP calculation using the above-described (quasi-)relativistic ECPs. Including the SOC-induced differential stabilization, Δ*E*^SOC^ = *E*^SOC^(Sm(III)) – *E*^SOC^(Sm(II)), of the Sm(III) versus the Sm(II) ground state, the reaction free energy in THF was calculated using eq 2.
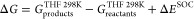
2

The corresponding reaction free energy in the gas phase (limited to the BDFE for SmI_2_(H_2_O)_5_ reported in Table S6) was calculated using eq 3.
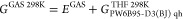
3*E*^GAS^ is the potential energy resulting from an SP calculation with a given method without applying the SMD solvation model. The reaction free energy (Δ*G*) was subsequently calculated using eq 4.
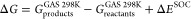
4

## Results and Discussion

### SOC Effects

SOC is a relativistic phenomenon which increases in importance with the nuclear charge. Since our computational models for estimation of the energy (*E*^THF^) of Sm(II) and Sm(III) complexes in THF solution only include scalar relativistic effects via quasi-relativistic ECPs, we here estimate the stabilization, expressed as a negative *E*^SOC^, of the ground states of these complexes induced by SOC.

As seen in Table 1, the SOC effect on the ground-state energy of Sm^2+^ and Sm^3+^ ions is mildly underestimated in the calculations compared to those taken as the difference between the ^7^F_0_ and ^6^H_5/2_^o^ ground states, respectively, and the average levels of the corresponding ^7^F and ^6^H^o^ terms of the atomic spectra.^54^ More importantly, the differential stabilization of Sm^3+^ versus Sm^2+^ is well reproduced, with  being overestimated by only ca. 0.5 kcal/mol. Also important, whereas the inclusion of solvent effects and ligands reduces the SOC-induced stabilization of the Sm(II) and Sm(III) ground states, Δ*E*^SOC^ appears to be little affected by the environment of the Sm^n+^ ions. Only minor increases in  are obtained on the inclusion of continuum solvation effects (for THF) and coordinating iodide, water, and hydroxide ligands in the calculations. Limited environmental influence on the SOC-induced stabilization is also indicated by the levels of the Sm^3+^^6^H^o^ term observed in various solvents^55,56^ being comparable to those of Sm^3+^ ions in LaCl_3_.^54^

**Table 1:** SOC-Induced Stabilization of Sm(II) and Sm(III) Ground States

system	calc/expt.	*E*^SOC^ [cm^–1^]	Δ*E*^SOC^ [kcal/mol]
Sm^2+^ free ion	expt.^a^	–2503.99	
Sm^3+^ ion in LaCl_3_	expt.^a^	–3761.21	–3.59
Sm^2+^ free ion	calc.	–2234.97	
Sm^3+^ free ion	calc.	–3680.29	–4.13
Sm^2+^ ion in THF	calc.^b^	–2232.27	
Sm^3+^ ion in THF	calc.^b^	–3682.18	–4.15
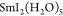 in THF	calc.^b^	–2050.50	
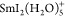 in THF	calc.^b^	–3547.44	–4.28
SmI_2_(H_2_O)_4_OH in THF	calc.^b^	–3550.52	–4.29

a From the spectrum of the free Sm^2+^ ion or from that of Sm^3+^ in LaCl_3_.^54^

b From calculations including continuum solvation effects of THF via the SMD model.

In conclusion, due to the limited influence of the environment on the SOC ground-state stabilization, we adopt the stabilization derived from the atomic spectra of Sm^2+^ and Sm^3+^ ions. In other words, Δ*E*^SOC^ = −3.59 kcal/mol (product: Sm(III)) or Δ*E*^SOC^ = 3.59 kcal/mol (product: Sm(II)) has been used in eqs 2 and 4 to correct relative free energies calculated using quasi-relativistic ECPs (parameterized to account for scalar relativistic effects) for SOC-induced differential stabilization of Sm(III) versus Sm(II).

### Reduction of SmI_2_^+^

First, the most straightforward test that any method applied to the study of reactions involving SmI_2_ as a reductant can be subjected to is to predict the corresponding one-electron reduction potential. The experimental reduction potential of SmI_2_^+^ has been reported to be −1.41 V versus Fc^+^/Fc (Fc = ferrocene) in THF at room temperature,^57^ which is equivalent to −0.78 V versus NHE (NHE = normal hydrogen electrode).^58,59^ Thus, the absolute reduction potential (*E*_abs_^0^) in THF is given by eq 5.^60,61^
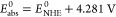
5

Consequently, *E*_abs_^0^ = 3.50 V for SmI_2_^+^, and the corresponding Δ*G*_red_^0^ of the reduction can be obtained from eq 6.
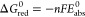
6where *n* is the number of electrons transferred, and *F* is the Faraday constant. Thus, the experimentally determined one-electron reduction potential of SmI_2_^+^ corresponds to a reaction free energy of −80.7 kcal/mol, which is the estimate against which reduction free energies calculated for SmI_2_(THF)_5_^+^/SmI_2_(THF)_5_ are compared (Table 2; additional results, including reduction potentials, in Table S4).

**Table 2:** Calculated Energetics and Natural Electron Populations (N) and Charges (q) of the Reduction of SmI_2_(THF)_5_^+^ to SmI_2_(THF)_5_ in THF^a^

				SmI_2_(THF)_5_^+^			SmI_2_(THF)_5_		
entry	method^b^	% HF exchange	Δ*G*^c^ [kcal/mol]	N (4f)	N (5d)	q (Sm)	N (4f)	N (5d)	q (Sm)
1	HF	100	–48.3	5.01	0.74	2.01	6.00	0.34	1.53
2	LSDA	0	–92.3	5.41	0.96	1.36	5.98	0.52	1.31
3	PBE^d^	0	–92.3	5.37	0.93	1.44	5.98	0.48	1.36
4	PBE-D3(BJ)^d^	0	–87.5	5.37	0.93	1.44	5.98	0.48	1.36
5	M06L-D3	0	–83.0	5.30	0.92	1.52	5.98	0.47	1.39
6	TPSS-D3(BJ)^e^	0	–82.6	5.32	0.93	1.50	5.98	0.47	1.38
7	PW6B95-D3(BJ)	28	–89.9	5.09	0.95	1.70	5.99	0.43	1.41
8	B3LYP-D3(BJ)	20	–86.7	5.13	0.93	1.68	5.99	0.43	1.42
9	M06-D3	27	–104.3	5.24	0.91	1.59	5.99	0.46	1.39
10	M062X-D3	54	–99.8	5.04	0.90	1.78	6.00	0.43	1.41
11	M06HF-D3	100	–82.3	5.04	0.83	1.89	6.00	0.40	1.45
12	PBEQIDH-D3(BJ)	69.3	–78.1	5.02^f^	0.94^f^	1.75^f^	5.97	0.43	1.42
13	DSD-PBEP86	69	–78.2	5.00	0.70	2.06	5.97	0.45	1.40
14	revDSD-PBEP86	69	–75.7	5.01	0.70	2.06	5.98	0.45	1.41
15	B2PLYP-D3(BJ)	53	–78.6	5.03	0.97	1.71	5.97	0.45	1.40
16	expt.^g^		–80.7						

a Energies and properties obtained in SP calculations using the SMD continuum solvation model for the THF solvent on geometries optimized using the PW6B95-D3(BJ) functional. Population analysis was carried out according to the NPA/NBO scheme.

b See the Supporting Information for the definition of the methods.

c Calculated using eq 2.

d Combination of the PBE exchange functional and the PBE correlation.

e Combination of the TPSS exchange functional and the TPSS correlation.

f Calculated using the “FixDM” keyword.

g Calculated from ref (57) using eqs 5 and 6.

As expected for an electron uptake, the estimated reaction energy is very sensitive to the correlation treatment. Whereas Hartree–Fock, lacking electron correlation, underestimates the stability of the reduced neutral complex by almost 33 kcal/mol (entry 1, Table 2), the DFAs overestimate the exergonicity, or equivalently, the reduction potential, to varying degrees. Still, compared to HF, even standard first- and second-rung functionals (entry 2–4) roughly halve the errors. Accounting for dispersion stabilizes the more compact SmI_2_(THF)_5_^+^ complex (the average Sm–O(THF) distance is 0.15 Å shorter than in SmI_2_(THF)_5_; see Figure S1 for optimized geometries) and cuts the overestimation of the exergonicity to ca. 7 kcal/mol (entry 4). Including dependency on the kinetic energy density in the exchange–correlation functional (third rung) reduces the error by another 4–5 kcal/mol (entry 5–6).

In contrast, including moderate components of exact (HF) exchange, as in popular, dispersion-including, fourth-rung functionals such as B3LYP-D3(BJ), M06-D3, and M062X-D3 (entry 8–10), and various range-separated functionals (Table S4), invariably leads to larger errors. A significant improvement over the third rung is only seen when large components of exact (HF) exchange are included. The effect of HF exchange is particularly striking for the hybrid Minnesota functionals (entry 9–11). M06-D3 and M062X-D3 overestimate the stability of the neutral complex relative to the cationic complex by almost 23.6 and 19.1 kcal/mol, respectively. Only on the inclusion of 100% HF exchange is this overestimation reduced to below 2 kcal/mol (M06HF-D3, entry 11). The improvements resulting from terms involving kinetic-energy density and from large components of HF exchange are diagnostic of the DE (which includes the self-interaction error) of DFAs.^62^ The DE may all but disappear when combining HF exchange with second-order perturbative correlation treatment.^62^ Indeed, whereas the regular, DE-suffering DFAs all overestimate the exergonicity of the reduction, the double-hybrid methods undershoot, but not by much. Except for revDSD-PBEP86^63^ (off by 5 kcal/mol), they are within 3 kcal/mol of the experiment (entry 12–15).

The magnitude of the DE for lanthanide complexes has been found in detailed work by Duignan and Autschbach^64^ to correlate with the lanthanide 4f and 5d electron populations. The likely explanation for this correlation is that the DE leads to artificial mixing of metal and ligand orbitals, excess ligand-to-metal electron donation (i.e., delocalization), and exaggerated 4f and 5d populations.^64^ Indeed, here the DE-free HF and presumably DE-free double-hybrid methods predict the combined 4f and 5d populations of SmI_2_(THF)_5_^+^ to be 0.30–0.60 lower than those of standard GGA-based DFAs (cf., entry 4 and 12–15, Table 2), resulting in a more positively charged Sm center.

### O–H BDFE of SmI_2_(THF)_4_H_2_O

Having identified methods predicting the energetics of the SmI_2_(THF)_5_^+^/SmI_2_(THF)_5_ reduction with excellent accuracy and little interference from the DE, we next turned to a PCET-relevant reaction for which the energetics can be expected to depend on the Sm(II)/Sm(III) relative stability: the rupture of a O–H bond of SmI_2_(THF)_4_H_2_O to give SmI_2_(THF)_4_OH and H^•^. The BDFE of PCET reactions can be estimated from experimental parameters using eq 7.^65^
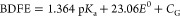
7

Here, to estimate the BDFE resulting from mixing 1 equiv of water with SmI_2_ in THF solution, we note that the most relevant p*K*_a_ available is that of SmI_2_–H_2_O in water, reported to be 7.11.^66^ Similarly, the most applicable reduction potential *E*^0^ is that for SmI_2_ in THF, which is −1.41 V versus Fc^+^/Fc.^57^ We assume negligible changes in *E*^0^ on the replacement of a THF by a water molecule. Next, *C*_G_ is the free energy of H^+^/H_2_ reduction, reported to be 60.4 kcal/mol in THF.^66^ Thus, the BDFE of the PCET conducted by SmI_2_(THF)_4_H_2_O in THF can be estimated to 37.6 kcal/mol. Lower estimates have been made for more water-rich mixtures,^67^ but excess water increases the reductive power^2,68^ and thereby lowers the BDFE, of SmI_2_. For the present 1:1 water/SmI_2_ mixture, eq 7 in conjunction with the above experimental information is assumed to offer the best estimate of our PCET-relevant BDFE (37.6 kcal/mol). This is thus the estimate against which a selection of BDFEs calculated using methods of rung 3–5 for SmI_2_(THF)_4_H_2_O are compared (Table 3; additional results in Table S5).

**Table 3:** O–H BDFEs of SmI_2_(THF)_4_H_2_O in THF^a^

entry	method^b^	BDFE^c^ [kcal/mol]
1	M06L-D3	49.6
2	TPSS-D3(BJ)	53.4
3	PW6B95-D3(BJ)	59.8
4	M06HF-D3	51.0
5	PBEQIDH-D3(BJ)	36.2
6	DSD-PBEP86	37.3
7	B2PLYP-D3(BJ)	45.4
8	expt.^d^	37.6

a See Table S5 for additional calculated BDFEs.

b See Table S2 for definitions of the methods.

c Calculated using eq 2.

d Obtained from experimental parameters using eq 7.

As for the above reduction free energies, the best-performing methods of Table 3 tend to stabilize Sm(III) versus Sm(II), thereby predicting lower BDFEs. However, as already indicated by the presence of components other than *E*^0^ in eq 7, the BDFE is harder to predict. DFAs estimating the reduction free energy to within 2–3 kcal/mol, such as M06L-D3 and M06HF-D3, are, with errors of 12–13 kcal/mol, unsuitable for predicting the BDFE. To analyze the physical origins of these errors, we note that an O–H σ-bond and a dative Sm–OH_2_ bond are replaced by a short, polar Sm–OH bond (2.15 Å, 9 pm shorter than the minimum Sm–O distance in a distribution of bond distances between Sm^3+^ and O^2–^ ions determined by X-ray crystallography).^69^ The close Sm–O contact suggests that correlation effects of the Sm(III) state influence the BDFE more than the SmI_2_(THF)_5_^+^/SmI_2_(THF)_5_ reduction potential. This is likely the reason why mixed performance is observed even among double-hybrid methods. Combined with empirical dispersion corrections (D3(BJ)), B2PLYP, one of the first double-hybrid methods to be suggested,^70^ predicts a BDFE that is almost 8 kcal/mol too high, whereas subsequently developed double-hybrid methods (DSD-PBEP86,^71^ and PBEQIDH-D3(BJ)),^72,73^ consistent with their improved performance in validation studies,^74,75^ are within 2 kcal/mol of the experimental estimate. As for the above one-electron reduction (Table 2), the reparametrized DSD-PBEP86 functional, revDSD-PBEP86,^63^ overestimates the stability of the +III versus the +II oxidation state of Sm and predicts a BDFE that is too low by almost 4 kcal/mol (Table S5). Still, even with this spread among the double-hybrid methods, we note that they all predict the BDFE to within 8 kcal/mol of the experiment and that their average prediction (38.2 kcal/mol) is within 1 kcal/mol. In contrast, the best DFAs of rungs three and four all overshoot the BDFE by at least 10 kcal/mol, and many popular hybrid DFAs, such as the long-range-corrected ωB97X-D functional^76^ (Table S5), are associated with errors on the order of 20 kcal/mol.

### O–H BDFE of SmI_2_(H_2_O)_5_

To test whether the above agreement between the BDFE predicted by the best double-hybrid methods and the experiment might be the result of a fortuitous cancellation of errors, for example, involving continuum-model solvent effects, corresponding BDFEs were also calculated for the model complex SmI_2_(H_2_O)_5_, the geometry of which was optimized starting from the Sm and O positions of SmI_2_(THF)_4_H_2_O (Figure S1). For the small water-based model complex, BDFE could be obtained using the coupled-cluster CCSD(T) method,^77^ which involves single and double substitutions of the HF reference along with a perturbative estimate of connected triples. Thus, the water-based model complex allowed for comparing gas-phase DFT-predicted BDFEs directly with those obtained using CCSD(T), thereby circumventing the impact of the solvent model in the validation. Other uncertainties are also cancelled out, such as those associated with the SOC-induced differential stabilization of the Sm^n+^ ground states: all the BDFEs calculated for SmI_2_(H_2_O)_5_ have been corrected with the same standard correction (Δ*E*^SOC^ = −3.59 kcal/mol) to account for SOC stabilizing Sm(III) more than Sm(II).

Before proceeding to the gas-phase calculations, we note that the BDFE predicted for SmI_2_(H_2_O)_5_ in THF is lower (by 6 kcal/mol, cf. entry 2 in Table 4 vs entry 3 in Table 3) than that for SmI_2_(THF)_4_H_2_O, consistent with the observed increased reducing power and lowering of the BDFE on addition of water to solutions of SmI_2_.^2,68^ Removing the continuum solvent treatment further lowers the BDFE by 3.3 kcal/mol (entry 3, Table 4). Consequently, the CCSD(T)-predicted BDFE for SmI_2_(H_2_O)_5_ in the gas phase (28.3 kcal/mol, entry 8 in Table 4) is lower than the experimental estimate for SmI_2_(THF)_4_H_2_O in THF (37.6 kcal/mol, entry 8 in Table 3). With the T1 diagnostic being well below 0.02^78^ for both SmI_2_(H_2_O)_5_ (0.0113) and SmI_2_(H_2_O)_4_OH (0.0124), the CCSD(T)-predicted BDFE is here taken as the value against which the methods of Table 3 are compared. Moreover, the low T1 values indicate that neither Sm(II) nor Sm(III) is heavily influenced by nondynamical correlation and that most of the correlation effects may be recovered perturbatively.

**Table 4:** O–H BDFEs of SmI_2_(H_2_O)_5_ in the Gas Phase^a^

entry	method^b^	BDFE^c^ [kcal/mol]
1	M06L-D3	41.7
2	PW6B95-D3(BJ)^d^	53.8
3	PW6B95-D3(BJ)	48.7
4	M06HF-D3	44.9
5	PBEQIDH-D3(BJ)	35.7
6	DSD-PBEP86	28.8
7	CCSD	29.1
8	CCSD(T)	28.3

a See Table S6 for additional calculated BDFEs.

b See Table S2 for definitions of the methods.

c Calculated using eq 4.

d Including THF solvent effects via the SMD continuum solvation model.

This is confirmed by the double-hybrid methods, which, as for Table 3 above, are alone in predicting BDFEs within 10 kcal/mol of CCSD(T) and in being reasonably well centered around this reference. The methods with the smallest deviation from CCSD(T) are the two double hybrids that include spin-component scaling of the MP2-like correlation, DSD-PBEP86 and revDSD-PBEP86 (Table S6). The performance of the original DSD-PBEP86 functional is particularly impressive, with both BDFEs being within 1 kcal/mol of the reference (Tables 3 and 4) and the reduction free energy (Table 2) being within 3 kcal/mol. The spin-component scaling enables, via parametrization, more of the correlation effects to be captured, and the two DSD functionals are also the methods with the highest reported general accuracy among the double-hybrid methods used here.^74^ It seems plausible that spin-component scaling better captures the correlation effects of the compact Sm(III) water complex, in which the Sm–O bond (2.11 Å) is even shorter than that of SmI_2_(THF)_4_OH (2.15 Å). Finally, the highly accurate relative free energies predicted here by DSD-PBEP86, in particular, together with those of a challenging C–C bond forming reaction initiated by SET from SmI_2_,^8^ suggest that DSD-PBEP86 and other spin-component-scaled and parametrized double-hybrid methods might represent the “simple and effective” strategy called for^100^ to describe the redox and SET chemistry of SmI_2_.

## Conclusions

The true density functionals, that is, DFAs depending only on the density or quantities derived directly from the density, tested here are unsuitable for predicting energetics of redox and PCET processes of SmI_2_ when only accounting for scalar relativistic effects. Fortunately, the differential SOC-induced stabilization of the Sm(III) versus the Sm(II) ground state appears to be little influenced by ligands and solvent. Correcting the relative energies with a standard differential (Δ*E*^SOC^) derived from atomic spectra improves the agreement with the experiment and brings a couple of common meta-GGAs (M06L-D3 and TPSS-D3(BJ)) and a hybrid meta-GGA functional with 100% exact exchange (M06HF-D3) to within 5 kcal/mol of the experimental reduction free energy of the Sm(III)/Sm(II) redox couple. Still, even with SOC corrections, the most accurate functionals overshoot O–H BDFEs of SmI_2_-induced PCET reactions by more than 10 kcal/mol, with many popular GGAs and hybrid-GGA functionals predicting BDFEs that are 15–20 kcal/mol too high. Most of this failure of regular DFAs is caused by the DE, which leads to excess ligand-to-metal electron donation and destabilizes the +III versus the +II oxidation state of Sm. Fortunately, the present systems do not appear to be heavily influenced by nondynamical correlation effects, and including information from virtual orbitals via perturbation theory, as in double-hybrid methods, reduces the DE and improves the predicted energetics significantly. In particular, the high accuracy obtained here for DSD-PBEP86 is promising with respect to the use of this and other spin-component scaled and parametrized double-hybrid methods in future studies of the rich redox-related chemistry of SmI_2_.

## Supporting Information Available

Additional computational details and results (PDF)
Cartesian coordinates of optimized complexes (XYZ)

## Supplementary Material

jp3c00418_si_001.pdf

jp3c00418_si_002.xyz
