# Supplementary material for: Enabling Molecular-Level Computational Description of Redox and Proton-Coupled Electron Transfer Reactions of Samarium Diiodide
Source: J Phys Chem A. 2023 Apr 19;127(17):3796–803. doi: 10.1021/acs.jpca.3c00418 (PMC10165656; doi:10.1021/acs.jpca.3c00418)
Supplement: Supplementary file 1 — jp3c00418_si_001.pdf [file jp3c00418_si_001.pdf]

SUPPORTING INFORMATION:

ENABLING MOLECULAR-LEVEL COMPUTATIONAL DESCRIPTION OF REDOX AND PROTON-COUPLED ELECTRON TRANSFER REACTIONS OF SAMARIUM DIIODIDE

Jonas Himmelstrup, Vidar R. Jensen*

Department of Chemistry, University of Bergen, NO-5007 Bergen, Norway

E-mail: Vidar.Jensen@uib.no

Table of Contents

S1: Additional Computational Details S2
S1.1: Basis sets used for Geometry Optimizations S2
S1.2: Overview of the Methods and basis sets used for SP Calculations S2
S1.3: Sample Input files S3
S2: Additional Computational results S19
S2.1: Evaluation of optimized geometries and selected bond lengths S19
S2.2: Reaction Potentials and Free energies S20
S2.3: Determination of Basis set superposition errors S21
S2.4: Determination of the influence of spin orbit coupling S22
S3: References S23

S1: ADDITIONAL COMPUTATIONAL DETAILS

S1.1: BASIS SETS USED FOR GEOMETRY OPTIMIZATIONS

Table S1 contains an overview of the basis sets used for geometry optimization.

Table S1: Overview of Basis Sets Used for Geometry Optimization.

Atom	Basis set acronym	Primitives	Contracted	ECP	References
H	cc-pVDZ	(4s, 1p)	[2s, 1p]	-	[1]
C	cc-pVDZ	(8s, 3p, 1d)	[3s, 1p, 1d]	-	[1]
O	aug-cc-pVDZ	(9s, 4p, 2d)	[4s, 3p, 2d]	-	[1–5]
I	aug-cc-pVDZ-PP	(7s, 6p, 6d)	[5s, 4p, 3d]	ECP28MDF	[6,7]
Sm(III)<br>Sm(II)	-	(7s, 6p, 5d)	[5s, 4p, 3d]	ECP51MWB<br>ECP52MWB	[8,9]

S1.2: OVERVIEW OF THE METHODS AND BASIS SETS USED FOR SP CALCULATIONS

A range of different computational methods were used in the single-point (SP) calculations (Table S2).

Table S2: Overview of the Methods Used in the SP Calculations.

Rung	Method	References	Comments
-	HF	[10]	Hartree-Fock
1	LSDA	[11–13]	Local spin-density approximation
2	PBE	[14,15]	GGA functional
3	M06L	[16]	Meta-GGA functional
3	TPSS	[17]	Meta-GGA functional
4	PW6B95	[18]	Global hybrid functional
4	B3LYP	[19]	Global hybrid functional
4	M06	[20]	Global hybrid meta-GGA functional
4	M062X	[20]	Global hybrid meta-GGA functional
4	M06HF	[21,22]	Global hybrid meta-GGA functional
4	BHandHLYP	[23]	Global hybrid functional
4	CAM-B3LYP	[24]	Range-separated hybrid functional
4	ωB97XD	[25]	Range-separated hybrid functional
4	LC-ωHPBE	[26]	Range-separated hybrid functional
5	B2PLYP	[27]	Double-hybrid functional
5	DSD-PBEP86	[28]	Double-hybrid functional with spin component scaling, including empirical dispersion corrections (D3(BJ))

5	revDSD-PBEP86	[29]	Modified version of DSD-PBEP86 including empirical dispersion corrections (D3(BJ))
5	PBEQIDH	[30]	Double-hybrid functional
-	D3	[31]	Dispersion corrections
-	D3(BJ)	[32–34]	Dispersion corrections with Becke-Johnson damping.
-	CCSD	[35–37]	Wave-function correlated method
-	CCSD(T)	[35,38]	Wave-function correlated method

An overview of the basis sets used in the SP calculations is given in Table S3.

Table S3: Overview of Basis Sets Used for Single-Point Calculations.

Atom	Basis set acronym	Primitives	Contracted	ECP	References
H	cc-pVTZ	(6s, 2p, 1d)	[3s, 2p, 1d]	-	[1]
C	cc-pVTZ	(8s, 3p, 2d, 1f)	[4s, 3p, 2d, 1f]	-	[1]
O	aug-cc-pVTZ	(9s, 4p, 3d, 2f)	[5s, 4p, 3d, 2f]	-	[1–5]
I	aug-cc-pVTZ-PP	(13s, 12p, 8d, 2f)	[6s, 5p, 4d, 2f]	ECP28MDF	[6,7]
Sm	-	(14s, 13p, 10d, 8f, 6g)	[10s, 8p, 5d, 4f, 3g]	ECP28MWB	[39,40]

S1.3: SAMPLE INPUT FILES

Sample input file for geometry optimization: optimization of $SmI_2(THF)_5$. The input file reflects a compound job including initial single-point SCF calculations and stability test to ensure convergence to a stable wavefunction.

```
%CHK=SmI2thf5
#P
# HF/GENECP 5D 7F
# NOSYMM
# SCF=(VSHIFT=1000,NOSYM,NOVARACC,MAXCYCLE=180,CONVER=4)
# SP

 Step 1 - Protocol JHI-2.0

0 1
Sm       -0.000100000000        0.002300000000        0.000200000000
I        0.015500000000       -0.018400000000        3.314900000000
I       -0.015600000000       -0.020300000000       -3.314600000000
O       -0.001200000000       -2.665600000000        0.000900000000
C        0.559600000000       -3.501300000000       -1.052200000000
C       -0.562700000000       -3.500200000000        1.054600000000
C        0.037100000000       -4.918400000000       -0.769200000000
H        0.244300000000       -3.089200000000       -2.014900000000
H        1.653400000000       -3.462900000000       -0.978700000000
C       -0.041300000000       -4.917900000000        0.772500000000
H       -0.247000000000       -3.087700000000        2.017000000000
H       -1.656500000000       -3.461000000000        0.981200000000
H        0.701200000000       -5.690900000000       -1.166900000000
H       -0.964200000000       -5.046100000000       -1.197300000000
H        0.959900000000       -5.046100000000        1.200700000000
H       -0.706000000000       -5.689600000000        1.170600000000
O       -2.530200000000       -0.830100000000        0.010300000000
C       -3.143500000000       -1.648100000000       -1.029000000000
C       -3.504900000000       -0.528400000000        1.046400000000
C       -4.645400000000       -1.687300000000       -0.695200000000
H       -2.913600000000       -1.197500000000       -1.998600000000
H       -2.698200000000       -2.648500000000       -0.978700000000
C       -4.632900000000       -1.549400000000        0.842600000000
H       -2.998000000000       -0.602100000000        2.012800000000
H       -3.872300000000        0.495200000000        0.898500000000
H       -5.119200000000       -2.610400000000       -1.040600000000
```

```
H       -5.158500000000       -0.829200000000       -1.144800000000
H       -4.364300000000       -2.505700000000        1.306500000000
H       -5.586200000000       -1.202900000000        1.251200000000
O       -1.565100000000        2.159300000000       -0.008800000000
C       -2.498800000000        2.517300000000       -1.063900000000
C       -1.596200000000        3.166200000000        1.045800000000
C       -2.745300000000        4.020100000000       -0.878900000000
H       -2.038700000000        2.247200000000       -2.018600000000
H       -3.428400000000        1.949500000000       -0.926200000000
C       -2.722700000000        4.143900000000        0.660300000000
H       -1.757100000000        2.659100000000        2.001400000000
H       -0.620700000000        3.664600000000        1.061100000000
H       -3.690600000000        4.344900000000       -1.322200000000
H       -1.920800000000        4.593400000000       -1.318800000000
H       -3.679500000000        3.807400000000        1.076300000000
H       -2.519500000000        5.161000000000        1.006900000000
O        1.566700000000        2.158200000000        0.007500000000
C        2.500600000000        2.516300000000        1.062300000000
C        1.598900000000        3.164200000000       -1.048000000000
C        2.749000000000        4.018600000000        0.875700000000
H        2.040100000000        2.247800000000        2.017300000000
H        3.429500000000        1.947100000000        0.925500000000
C        2.726700000000        4.140800000000       -0.663600000000
H        1.758900000000        2.656000000000       -2.003200000000
H        0.624000000000        3.663800000000       -1.063600000000
H        3.694800000000        4.342600000000        1.318700000000
H        1.925300000000        4.593400000000        1.315000000000
H        3.683000000000        3.802500000000       -1.079300000000
H        2.524800000000        5.157700000000       -1.011300000000
O        2.529400000000       -0.832100000000       -0.009300000000
C        3.504300000000       -0.531600000000       -1.045700000000
C        3.142100000000       -1.650100000000        1.030300000000
C        4.631400000000       -1.553500000000       -0.841500000000
H        2.997200000000       -0.605200000000       -2.011900000000
H        3.872600000000        0.491700000000       -0.898100000000
C        4.644000000000       -1.690700000000        0.696300000000
H        2.912800000000       -1.198800000000        1.999700000000
H        2.696000000000       -2.650200000000        0.980600000000
H        5.585000000000       -1.208000000000       -1.250400000000
H        4.361900000000       -2.509800000000       -1.305000000000
H        5.157900000000       -0.832900000000        1.145500000000
H        5.117000000000       -2.614100000000        1.042100000000

H     0
S   4   1.00
      1.301000D+01           1.968500D-02
      1.962000D+00           1.379770D-01
      4.446000D-01           4.781480D-01
      1.220000D-01           5.012400D-01
S   1   1.00
      1.220000D-01           1.000000D+00
P   1   1.00
      7.270000D-01           1.0000000
****
C     0
S   9   1.00
      6.665000D+03           6.920000D-04
      1.000000D+03           5.329000D-03
      2.280000D+02           2.707700D-02
      6.471000D+01           1.017180D-01
      2.106000D+01           2.747400D-01
      7.495000D+00           4.485640D-01
      2.797000D+00           2.850740D-01
      5.215000D-01           1.520400D-02
      1.596000D-01          -3.191000D-03
S   9   1.00
      6.665000D+03          -1.460000D-04
      1.000000D+03          -1.154000D-03
      2.280000D+02          -5.725000D-03
      6.471000D+01          -2.331200D-02
      2.106000D+01          -6.395500D-02
```

```
      7.495000D+00          -1.499810D-01
      2.797000D+00          -1.272620D-01
      5.215000D-01           5.445290D-01
      1.596000D-01           5.804960D-01
S   1   1.00
      1.596000D-01           1.000000D+00
P   4   1.00
      9.439000D+00           3.810900D-02
      2.002000D+00           2.094800D-01
      5.456000D-01           5.085570D-01
      1.517000D-01           4.688420D-01
P   1   1.00
      1.517000D-01           1.000000D+00
D   1   1.00
      5.500000D-01           1.0000000
****
O     0
S   9   1.00
      1.172000D+04           7.100000D-04
      1.759000D+03           5.470000D-03
      4.008000D+02           2.783700D-02
      1.137000D+02           1.048000D-01
      3.703000D+01           2.830620D-01
      1.327000D+01           4.487190D-01
      5.025000D+00           2.709520D-01
      1.013000D+00           1.545800D-02
      3.023000D-01          -2.585000D-03
S   9   1.00
      1.172000D+04          -1.600000D-04
      1.759000D+03          -1.263000D-03
      4.008000D+02          -6.267000D-03
      1.137000D+02          -2.571600D-02
      3.703000D+01          -7.092400D-02
      1.327000D+01          -1.654110D-01
      5.025000D+00          -1.169550D-01
      1.013000D+00           5.573680D-01
      3.023000D-01           5.727590D-01
S   1   1.00
      3.023000D-01           1.000000D+00
S   1   1.00
      0.0789600              1.0000000
P   4   1.00
      1.770000D+01           4.301800D-02
      3.854000D+00           2.289130D-01
      1.046000D+00           5.087280D-01
      2.753000D-01           4.605310D-01
P   1   1.00
      2.753000D-01           1.000000D+00
P   1   1.00
      0.0685600              1.0000000
D   1   1.00
      1.185000D+00           1.0000000
D   1   1.00
      0.3320000              1.0000000
****
I     0
S   8   1.00
      2.449790D+03           4.190000D-04
      3.598080D+02           2.240000D-03
      1.440580D+01           3.972230D-01
      9.076320D+00          -9.322490D-01
      2.088100D+00           9.371380D-01
      1.034980D+00           3.920860D-01
      3.162840D-01           1.248500D-02
      1.217190D-01          -1.329000D-03
S   8   1.00
      2.449790D+03           1.750000D-04
      3.598080D+02           1.057000D-03
      1.440580D+01           1.690000D-01
      9.076320D+00          -4.217930D-01
      2.088100D+00           6.388640D-01
```

```
      1.034980D+00           3.201150D-01
      3.162840D-01          -8.144280D-01
      1.217190D-01          -4.897980D-01
S   1   1.00
      3.162840D-01           1.000000D+00
S   1   1.00
      1.217190D-01           1.000000D+00
S   1   1.00
      4.200000D-02           1.0000000
P   6   1.00
      1.953010D+01           5.893400D-02
      1.108820D+01          -2.309300D-01
      2.715630D+00           6.648010D-01
      1.204300D+00           4.506730D-01
      3.399450D-01           2.898000D-02
      1.108810D-01          -2.889000D-03
P   6   1.00
      1.953010D+01          -1.883600D-02
      1.108820D+01           8.000600D-02
      2.715630D+00          -3.066520D-01
      1.204300D+00          -1.475940D-01
      3.399450D-01           6.075060D-01
      1.108810D-01           5.470490D-01
P   1   1.00
      1.108810D-01           1.000000D+00
P   1   1.00
      3.380000D-02           1.0000000
D   6   1.00
      4.547650D+01           4.266000D-03
      1.319280D+01          -1.362500D-02
      4.227410D+00           3.097560D-01
      1.942800D+00           5.097720D-01
      8.397710D-01           2.974610D-01
      3.000000D-01           4.016400D-02
D   1   1.00
      3.000000D-01           1.000000D+00
D   1   1.00
      1.191000D-01           1.0000000
****
Sm 0
S 3 1.00
7.0842110 -0.2887660
5.8425300 0.6989860
2.4631030 -1.3581220
S 1 1.00
0.5375410 1.0
S 1 1.00
0.2596980 1.0
S 1 1.00
0.0497100 1.0
S 1 1.00
0.0214080 1.0
P 3 1.00
4.3774220 0.2468220
2.8695050 -0.7075000
0.7160780 1.2334250
P 1 1.00
0.3113850 1.0
P 1 1.00
0.1115480 1.0
P 1 1.00
0.0317070 1.0
D 3 1.00
2.2921440 -0.0725130
0.7575830 0.3731340
0.3314430 0.7163430
D 1 1.00
0.1266380 1.0
D 1 1.00
0.0423830 1.0
****
```

```
I      0
I-ECP  4  28
g potential
  1
2 1.00000000E+00      0.00000000E+00
s-g potential
  3
2 4.003337600E+01      4.998964900E+01
2 1.730057600E+01      2.8100655600E+02
2 8.85172000E+00      6.141673900E+01
p-g potential
  4
2 1.572014100E+01      6.741623900E+01
2 1.520822200E+01      1.3480769600E+02
2 8.29418600E+00      1.456654800E+01
2 7.75394900E+00      2.896842200E+01
d-g potential
  4
2 1.381775100E+01      3.553875600E+01
2 1.358780500E+01      5.333975900E+01
2 6.94763000E+00      9.71646600E+00
2 6.96009900E+00      1.497750000E+01
f-g potential
  4
2 1.852295000E+01     -2.017661800E+01
2 1.825103500E+01     -2.608807700E+01
2 7.55790100E+00     -2.2043400E-01
2 7.59740400E+00     -2.2164600E-01
Sm      0
ECP52MWB  4  52
G-Komponente
  1
2 1.000000E+00      0.000000E+00
S-G
  2
2 4.831200E+00      1.53167043E+02
2 2.415600E+00     -8.592215E+00
P-G
  2
2 4.061300E+00      9.8845412E+01
2 2.030700E+00     -2.655907E+00
D-G
  2
2 2.708300E+00      4.1022141E+01
2 1.354200E+00     -1.292004E+00
F-G
  1
2 5.099400E+00     -5.8460194E+01

--Link1—-
%CHK=SmI2thf5
#P
# PW6B95D3/CHKBASIS
# GEOM=CHECK
# GUESS=READ
# NOSYMM
# INT=ULTRAFINE
# SCRF=(READ,SMD,SOLVENT=THF)
# SCF=(VSHIFT=1000,NOSYM,NOVARACC,MAXCYCLE=300)
# SP

 Step 2 - Protocol JHI-2.0

0 1

Surface=SAS

--Link1—
%CHK=SmI2thf5
#P
```

```
# PW6B95D3/CHKBASIS
# GEOM=CHECK
# GUESS=READ
# NOSYMM
# INT=ULTRAFINE
# SCRF=(READ,SMD,SOLVENT=THF)
# SCF=(VSHIFT=700,NOSYM,NOVARACC,MAXCYCLE=300)
# STABLE=(OPT,RUHF)

 Step 3 - Protocol JHI-2.0

0 1

Surface=SAS

--Link1--
%CHK=SmI2thf5
#P
# PW6B95D3/CHKBASIS
# GEOM=CHECK
# GUESS=READ
# NOSYMM
# INT=ULTRAFINE
# SCRF=(READ,SMD,SOLVENT=THF)
# SCF=(VSHIFT=700,NOSYM,MAXCYCLE=700,CONVER=9)
# CPHF=(GRID=FINEGRID)
# OPT=(RECALCFC=25,MAXCYCLES=1000,TIGHT)

 Step 4 - Protocol JHI-2.0

0 1

Surface=SAS

--Link1--
%CHK=SmI2thf5
#P
# PW6B95D3/CHKBASIS
# GEOM=CHECK
# GUESS=READ
# NOSYMM
# INT=ULTRAFINE
# SCRF=(READ,SMD,SOLVENT=THF)
# SCF=(VSHIFT=700,NOSYM,MAXCYCLE=600,CONVER=9)
# CPHF=(GRID=FINEGRID)
# FREQ

 Step 5 - Protocol JHI-2.0

0 1

Surface=SAS
```

Sample file for SP calculations:

Input file for SP and NBO calculation on $SmI_2(THF)_5$ using the PW6B95-D3(BJ) functional. The Gaussian keyword PW6B95D3 invokes the PW6B95 functional combined with Grimme D3(BJ) empirical corrections.

```
%CHK=SmI2thf5
#P
# PW6B95D3/GENECP 5D 7F
# GEOM=CHECK
# GUESS=READ
# Pop=(NBO7Read)
# NOSYMM
# INT=ULTRAFINE
# SCRF=(SMD,SOLVENT=THF)
# SCF=(VSHIFT=300,NOSYM,MAXCYCLE=300,CONVER=5)
# SP

 Step 1 - Protocol JHI-2.0
```

```
0 7

H     0
S   5   1.00
      3.387000D+01          6.068000D-03
      5.095000D+00          4.530800D-02
      1.159000D+00          2.028220D-01
      3.258000D-01          5.039030D-01
      1.027000D-01          3.834210D-01
S   1   1.00
      3.258000D-01          1.000000D+00
S   1   1.00
      1.027000D-01          1.000000D+00
P   1   1.00
      1.407000D+00          1.000000D+00
P   1   1.00
      3.880000D-01          1.000000D+00
D   1   1.00
      1.057000D+00          1.0000000
****
C     0
S   10   1.00
      8.236000D+03          5.310000D-04
      1.235000D+03          4.108000D-03
      2.808000D+02          2.108700D-02
      7.927000D+01          8.185300D-02
      2.559000D+01          2.348170D-01
      8.997000D+00          4.344010D-01
      3.319000D+00          3.461290D-01
      9.059000D-01          3.937800D-02
      3.643000D-01         -8.983000D-03
      1.285000D-01          2.385000D-03
S   10   1.00
      8.236000D+03         -1.130000D-04
      1.235000D+03         -8.780000D-04
      2.808000D+02         -4.540000D-03
      7.927000D+01         -1.813300D-02
      2.559000D+01         -5.576000D-02
      8.997000D+00         -1.268950D-01
      3.319000D+00         -1.703520D-01
      9.059000D-01          1.403820D-01
      3.643000D-01          5.986840D-01
      1.285000D-01          3.953890D-01
S   1   1.00
      9.059000D-01          1.000000D+00
S   1   1.00
      1.285000D-01          1.000000D+00
P   5   1.00
      1.871000D+01          1.403100D-02
      4.133000D+00          8.686600D-02
      1.200000D+00          2.902160D-01
      3.827000D-01          5.010080D-01
      1.209000D-01          3.434060D-01
P   1   1.00
      3.827000D-01          1.000000D+00
P   1   1.00
      1.209000D-01          1.000000D+00
D   1   1.00
      1.097000D+00          1.000000D+00
D   1   1.00
      3.180000D-01          1.000000D+00
F   1   1.00
      7.610000D-01          1.0000000
****
O     0
S   10   1.00
      1.533000D+04          5.080000D-04
      2.299000D+03          3.929000D-03
      5.224000D+02          2.024300D-02
      1.473000D+02          7.918100D-02
      4.755000D+01          2.306870D-01
```

```
      1.676000D+01           4.331180D-01
      6.207000D+00           3.502600D-01
      1.752000D+00           4.272800D-02
      6.882000D-01          -8.154000D-03
      2.384000D-01           2.381000D-03
S   10   1.00
      1.533000D+04          -1.150000D-04
      2.299000D+03          -8.950000D-04
      5.224000D+02          -4.636000D-03
      1.473000D+02          -1.872400D-02
      4.755000D+01          -5.846300D-02
      1.676000D+01          -1.364630D-01
      6.207000D+00          -1.757400D-01
      1.752000D+00           1.609340D-01
      6.882000D-01           6.034180D-01
      2.384000D-01           3.787650D-01
S   1   1.00
      1.752000D+00           1.000000D+00
S   1   1.00
      2.384000D-01           1.000000D+00
S   1   1.00
      0.0737600              1.0000000
P   5   1.00
      3.446000D+01           1.592800D-02
      7.749000D+00           9.974000D-02
      2.280000D+00           3.104920D-01
      7.156000D-01           4.910260D-01
      2.140000D-01           3.363370D-01
P   1   1.00
      7.156000D-01           1.000000D+00
P   1   1.00
      2.140000D-01           1.000000D+00
P   1   1.00
      0.0597400              1.0000000
D   1   1.00
      2.314000D+00           1.000000D+00
D   1   1.00
      6.450000D-01           1.000000D+00
D   1   1.00
      0.2140000              1.0000000
F   1   1.00
      1.428000D+00           1.0000000
F   1   1.00
      0.5000000              1.0000000
****
I     0
S   11   1.00
      5.546500D+03           1.560000D-04
      8.382140D+02           9.860000D-04
      1.821870D+02           2.792000D-03
      3.121230D+01          -4.325100D-02
      1.953140D+01           2.341340D-01
      8.240990D+00          -7.509430D-01
      2.194550D+00           8.829680D-01
      1.109110D+00           4.620610D-01
      3.746410D-01           2.228600D-02
      1.770800D-01          -4.353000D-03
      8.106100D-02           1.102000D-03
S   11   1.00
      5.546500D+03          -7.300000D-05
      8.382140D+02          -5.080000D-04
      1.821870D+02          -1.158000D-03
      3.121230D+01           1.219300D-02
      1.953140D+01          -8.785400D-02
      8.240990D+00           3.382000D-01
      2.194550D+00          -5.765500D-01
      1.109110D+00          -4.092980D-01
      3.746410D-01           5.674590D-01
      1.770800D-01           6.124890D-01
      8.106100D-02           1.432310D-01
S   1   1.00
```

```
      3.746410D-01           1.000000D+00
S   1   1.00
      1.770800D-01           1.000000D+00
S   1   1.00
      1.001000D-01           1.000000D+00
S   1   1.00
      4.120000D-02           1.0000000
P   9   1.00
      1.889880D+02           5.850000D-04
      2.128680D+01           3.692300D-02
      1.003960D+01          -2.353240D-01
      3.451800D+00           3.414830D-01
      1.974560D+00           5.347880D-01
      1.024200D+00           2.651410D-01
      4.494370D-01           2.578700D-02
      1.866480D-01           5.220000D-04
      7.348100D-02           6.060000D-04
P   9   1.00
      1.889880D+02          -2.560000D-04
      2.128680D+01          -1.168200D-02
      1.003960D+01           8.319200D-02
      3.451800D+00          -1.569700D-01
      1.974560D+00          -2.245180D-01
      1.024200D+00          -1.144510D-01
      4.494370D-01           3.753560D-01
      1.866480D-01           5.751360D-01
      7.348100D-02           2.459170D-01
P   1   1.00
      5.981000D-01           1.000000D+00
P   1   1.00
      9.618000D-02           1.000000D+00
P   1   1.00
      3.590000D-02           1.0000000
D   9   1.00
      1.326620D+02           5.720000D-04
      3.760540D+01           4.402000D-03
      1.038910D+01          -4.092200D-02
      6.490170D+00           9.966100D-02
      3.454510D+00           3.226630D-01
      1.844130D+00           4.003430D-01
      9.624780D-01           2.683060D-01
      4.728530D-01           8.484700D-02
      1.932000D-01           7.632000D-03
D   1   1.00
      4.728530D-01           1.000000D+00
D   1   1.00
      1.932000D-01           1.000000D+00
D   1   1.00
      7.820000D-02           1.0000000
F   1   1.00
      4.064000D-01           1.000000D+00
F   1   1.00
      1.848000D-01           1.0000000
****
Sm     0
S   5   1.00
  70078.17100                0.000097
  10598.38400                0.000730
   2413.86600                0.003523
    677.40310                0.010873
    208.50730                0.017824
S   1   1.00
     39.36630                1.0
S   1   1.00
     27.98750                1.0
S   1   1.00
     14.34260                1.0
S   1   1.00
      3.61280                1.0
S   1   1.00
      1.84520                1.0
```

```
S   1   1.00
      0.68750                1.0
S   1   1.00
      0.30010                1.0
S   1   1.00
      0.05390                1.0
S   1   1.00
      0.02240                1.0
P   6   1.00
   3990.19500                0.000033
   1108.06210                0.000205
    385.81320                0.000983
    134.97630                0.003882
     28.61990                0.064056
     20.31750               -0.022684
P   1   1.00
     14.38990                1.0
P   1   1.00
      5.30930                1.0
P   1   1.00
      2.69080                1.0
P   1   1.00
      1.22020                1.0
P   1   1.00
      0.57260                1.0
P   1   1.00
      0.24670                1.0
P   1   1.00
      0.08000                1.0
D   6   1.00
    398.01540                0.000433
    115.54660                0.003852
     41.89370                0.017324
     20.77430                0.005743
      7.53040                0.259983
      4.04540                0.431478
D   1   1.00
      2.10120                1.0
D   1   1.00
      1.01050                1.0
D   1   1.00
      0.34750                1.0
D   1   1.00
      0.10830                1.0
F   5   1.00
    127.18630                0.003050
     45.93960                0.031145
     20.62340                0.113624
      9.38550                0.240128
      4.27900                0.347652
F   1   1.00
      1.88830                1.0
F   1   1.00
      0.77480                1.0
F   1   1.00
      0.27760                1.0
G   4   1.00
     20.62340                0.016405
      9.38550                0.067551
      4.27900                0.145472
      1.88830                0.353223
G   1   1.00
      0.77480                1.0
G   1   1.00
      0.27760                1.0
****

I-ECP     4     28
g potential
  1
2      1.00000000             0.00000000
```

```
s-g potential
  3
2     40.03337600           49.98964900
2     17.30057600          281.00655600
2      8.85172000           61.41673900
p-g potential
  4
2     15.72014100           67.41623900
2     15.20822200          134.80769600
2      8.29418600           14.56654800
2      7.75394900           28.96842200
d-g potential
  4
2     13.81775100           35.53875600
2     13.58780500           53.33975900
2      6.94763000            9.71646600
2      6.96009900           14.97750000
f-g potential
  4
2     18.52295000          -20.17661800
2     18.25103500          -26.08807700
2      7.55790100           -0.22043400
2      7.59740400           -0.22164600
SM     0
SM-ECP     5     28
h potential
  1
2      1.000000000            0.000000000
s-h potential
  1
2     22.344471000          572.985332000
p-h potential
  1
2     16.694590000          272.359145000
d-h potential
  1
2     13.727705000          115.293900000
f-h potential
  1
2     24.059092000          -51.108392000
g-h potential
  1
2     20.197249000          -25.421885000

$NBO FILE=FILE archive bndidx nbo DMNAO $END
```

Input file for SP and NBO calculation on $SmI_2(THF)_5$ using the double-hybrid functional DSDPBEP86, which includes empirical dispersion corrections.

```
%CHK=SmI2thf5
#P
# DSDPBEP86/GENECP 5D 4F
# Window=(43,0)
# GEOM=CHECK
# GUESS=READ
# Pop=(NBO7Read)
# Density=current
# NOSYMM
# INT=ULTRAFINE
# SCRF=(SMD,SOLVENT=THF)
# SCF=(VSHIFT=300,NOSYM,MAXCYCLE=1300,TIGHT)
# CPHF=(GRID=FINEGRID)
# SP

 Step 1 - Protocol JHI-2.0

0 7
```

```
H     0
S   5   1.00
      3.387000D+01           6.068000D-03
      5.095000D+00           4.530800D-02
      1.159000D+00           2.028220D-01
      3.258000D-01           5.039030D-01
      1.027000D-01           3.834210D-01
S   1   1.00
      3.258000D-01           1.000000D+00
S   1   1.00
      1.027000D-01           1.000000D+00
P   1   1.00
      1.407000D+00           1.000000D+00
P   1   1.00
      3.880000D-01           1.000000D+00
D   1   1.00
      1.057000D+00           1.0000000
****
C     0
S   10   1.00
      8.236000D+03           5.310000D-04
      1.235000D+03           4.108000D-03
      2.808000D+02           2.108700D-02
      7.927000D+01           8.185300D-02
      2.559000D+01           2.348170D-01
      8.997000D+00           4.344010D-01
      3.319000D+00           3.461290D-01
      9.059000D-01           3.937800D-02
      3.643000D-01          -8.983000D-03
      1.285000D-01           2.385000D-03
S   10   1.00
      8.236000D+03          -1.130000D-04
      1.235000D+03          -8.780000D-04
      2.808000D+02          -4.540000D-03
      7.927000D+01          -1.813300D-02
      2.559000D+01          -5.576000D-02
      8.997000D+00          -1.268950D-01
      3.319000D+00          -1.703520D-01
      9.059000D-01           1.403820D-01
      3.643000D-01           5.986840D-01
      1.285000D-01           3.953890D-01
S   1   1.00
      9.059000D-01           1.000000D+00
S   1   1.00
      1.285000D-01           1.000000D+00
P   5   1.00
      1.871000D+01           1.403100D-02
      4.133000D+00           8.686600D-02
      1.200000D+00           2.902160D-01
      3.827000D-01           5.010080D-01
      1.209000D-01           3.434060D-01
P   1   1.00
      3.827000D-01           1.000000D+00
P   1   1.00
      1.209000D-01           1.000000D+00
D   1   1.00
      1.097000D+00           1.000000D+00
D   1   1.00
      3.180000D-01           1.000000D+00
F   1   1.00
      7.610000D-01           1.0000000
****
O     0
S   10   1.00
      1.533000D+04           5.080000D-04
      2.299000D+03           3.929000D-03
      5.224000D+02           2.024300D-02
      1.473000D+02           7.918100D-02
      4.755000D+01           2.306870D-01
      1.676000D+01           4.331180D-01
      6.207000D+00           3.502600D-01
```

```
      1.752000D+00           4.272800D-02
      6.882000D-01          -8.154000D-03
      2.384000D-01           2.381000D-03
S   10   1.00
      1.533000D+04          -1.150000D-04
      2.299000D+03          -8.950000D-04
      5.224000D+02          -4.636000D-03
      1.473000D+02          -1.872400D-02
      4.755000D+01          -5.846300D-02
      1.676000D+01          -1.364630D-01
      6.207000D+00          -1.757400D-01
      1.752000D+00           1.609340D-01
      6.882000D-01           6.034180D-01
      2.384000D-01           3.787650D-01
S   1   1.00
      1.752000D+00           1.000000D+00
S   1   1.00
      2.384000D-01           1.000000D+00
S   1   1.00
      0.0737600              1.0000000
P   5   1.00
      3.446000D+01           1.592800D-02
      7.749000D+00           9.974000D-02
      2.280000D+00           3.104920D-01
      7.156000D-01           4.910260D-01
      2.140000D-01           3.363370D-01
P   1   1.00
      7.156000D-01           1.000000D+00
P   1   1.00
      2.140000D-01           1.000000D+00
P   1   1.00
      0.0597400              1.0000000
D   1   1.00
      2.314000D+00           1.000000D+00
D   1   1.00
      6.450000D-01           1.000000D+00
D   1   1.00
      0.2140000              1.0000000
F   1   1.00
      1.428000D+00           1.0000000
F   1   1.00
      0.5000000              1.0000000
****
I     0
S   11   1.00
      5.546500D+03           1.560000D-04
      8.382140D+02           9.860000D-04
      1.821870D+02           2.792000D-03
      3.121230D+01          -4.325100D-02
      1.953140D+01           2.341340D-01
      8.240990D+00          -7.509430D-01
      2.194550D+00           8.829680D-01
      1.109110D+00           4.620610D-01
      3.746410D-01           2.228600D-02
      1.770800D-01          -4.353000D-03
      8.106100D-02           1.102000D-03
S   11   1.00
      5.546500D+03          -7.300000D-05
      8.382140D+02          -5.080000D-04
      1.821870D+02          -1.158000D-03
      3.121230D+01           1.219300D-02
      1.953140D+01          -8.785400D-02
      8.240990D+00           3.382000D-01
      2.194550D+00          -5.765500D-01
      1.109110D+00          -4.092980D-01
      3.746410D-01           5.674590D-01
      1.770800D-01           6.124890D-01
      8.106100D-02           1.432310D-01
S   1   1.00
      3.746410D-01           1.000000D+00
S   1   1.00
```

```
      1.770800D-01           1.000000D+00
S   1   1.00
      1.001000D-01           1.000000D+00
S   1   1.00
      4.120000D-02           1.0000000
P   9   1.00
      1.889880D+02           5.850000D-04
      2.128680D+01           3.692300D-02
      1.003960D+01          -2.353240D-01
      3.451800D+00           3.414830D-01
      1.974560D+00           5.347880D-01
      1.024200D+00           2.651410D-01
      4.494370D-01           2.578700D-02
      1.866480D-01           5.220000D-04
      7.348100D-02           6.060000D-04
P   9   1.00
      1.889880D+02          -2.560000D-04
      2.128680D+01          -1.168200D-02
      1.003960D+01           8.319200D-02
      3.451800D+00          -1.569700D-01
      1.974560D+00          -2.245180D-01
      1.024200D+00          -1.144510D-01
      4.494370D-01           3.753560D-01
      1.866480D-01           5.751360D-01
      7.348100D-02           2.459170D-01
P   1   1.00
      5.981000D-01           1.000000D+00
P   1   1.00
      9.618000D-02           1.000000D+00
P   1   1.00
      3.590000D-02           1.0000000
D   9   1.00
      1.326620D+02           5.720000D-04
      3.760540D+01           4.402000D-03
      1.038910D+01          -4.092200D-02
      6.490170D+00           9.966100D-02
      3.454510D+00           3.226630D-01
      1.844130D+00           4.003430D-01
      9.624780D-01           2.683060D-01
      4.728530D-01           8.484700D-02
      1.932000D-01           7.632000D-03
D   1   1.00
      4.728530D-01           1.000000D+00
D   1   1.00
      1.932000D-01           1.000000D+00
D   1   1.00
      7.820000D-02           1.0000000
F   1   1.00
      4.064000D-01           1.000000D+00
F   1   1.00
      1.848000D-01           1.0000000
****
Sm     0
S   5   1.00
  70078.17100                0.000097
  10598.38400                0.000730
   2413.86600                0.003523
    677.40310                0.010873
    208.50730                0.017824
S   1   1.00
     39.36630                1.0
S   1   1.00
     27.98750                1.0
S   1   1.00
     14.34260                1.0
S   1   1.00
      3.61280                1.0
S   1   1.00
      1.84520                1.0
S   1   1.00
      0.68750                1.0
```

```
S   1   1.00
      0.30010                1.0
S   1   1.00
      0.05390                1.0
S   1   1.00
      0.02240                1.0
P   6   1.00
   3990.19500                0.000033
   1108.06210                0.000205
    385.81320                0.000983
    134.97630                0.003882
     28.61990                0.064056
     20.31750               -0.022684
P   1   1.00
     14.38990                1.0
P   1   1.00
      5.30930                1.0
P   1   1.00
      2.69080                1.0
P   1   1.00
      1.22020                1.0
P   1   1.00
      0.57260                1.0
P   1   1.00
      0.24670                1.0
P   1   1.00
      0.08000                1.0
D   6   1.00
    398.01540                0.000433
    115.54660                0.003852
     41.89370                0.017324
     20.77430                0.005743
      7.53040                0.259983
      4.04540                0.431478
D   1   1.00
      2.10120                1.0
D   1   1.00
      1.01050                1.0
D   1   1.00
      0.34750                1.0
D   1   1.00
      0.10830                1.0
F   5   1.00
    127.18630                0.003050
     45.93960                0.031145
     20.62340                0.113624
      9.38550                0.240128
      4.27900                0.347652
F   1   1.00
      1.88830                1.0
F   1   1.00
      0.77480                1.0
F   1   1.00
      0.27760                1.0
G   4   1.00
     20.62340                0.016405
      9.38550                0.067551
      4.27900                0.145472
      1.88830                0.353223
G   1   1.00
      0.77480                1.0
G   1   1.00
      0.27760                1.0
****

I-ECP     4     28
g potential
  1
2     1.00000000             0.00000000
s-g potential
  3
```

```
2     40.03337600           49.98964900
2     17.30057600          281.00655600
2      8.85172000           61.41673900
p-g potential
  4
2     15.72014100           67.41623900
2     15.20822200          134.80769600
2      8.29418600           14.56654800
2      7.75394900           28.96842200
d-g potential
  4
2     13.81775100           35.53875600
2     13.58780500           53.33975900
2      6.94763000            9.71646600
2      6.96009900           14.97750000
f-g potential
  4
2     18.52295000          -20.17661800
2     18.25103500          -26.08807700
2      7.55790100           -0.22043400
2      7.59740400           -0.22164600
SM     0
SM-ECP     5     28
h potential
  1
2      1.000000000            0.000000000
s-h potential
  1
2     22.344471000          572.985332000
p-h potential
  1
2     16.694590000          272.359145000
d-h potential
  1
2     13.727705000          115.293900000
f-h potential
  1
2     24.059092000          -51.108392000
g-h potential
  1
2     20.197249000          -25.421885000

$NBO FILE=FILE archive bndidx nbo DMNAO $END
```

S2: ADDITIONAL COMPUTATIONAL RESULTS

S2.1: EVALUATION OF OPTIMIZED GEOMETRIES AND SELECTED BOND LENGTHS

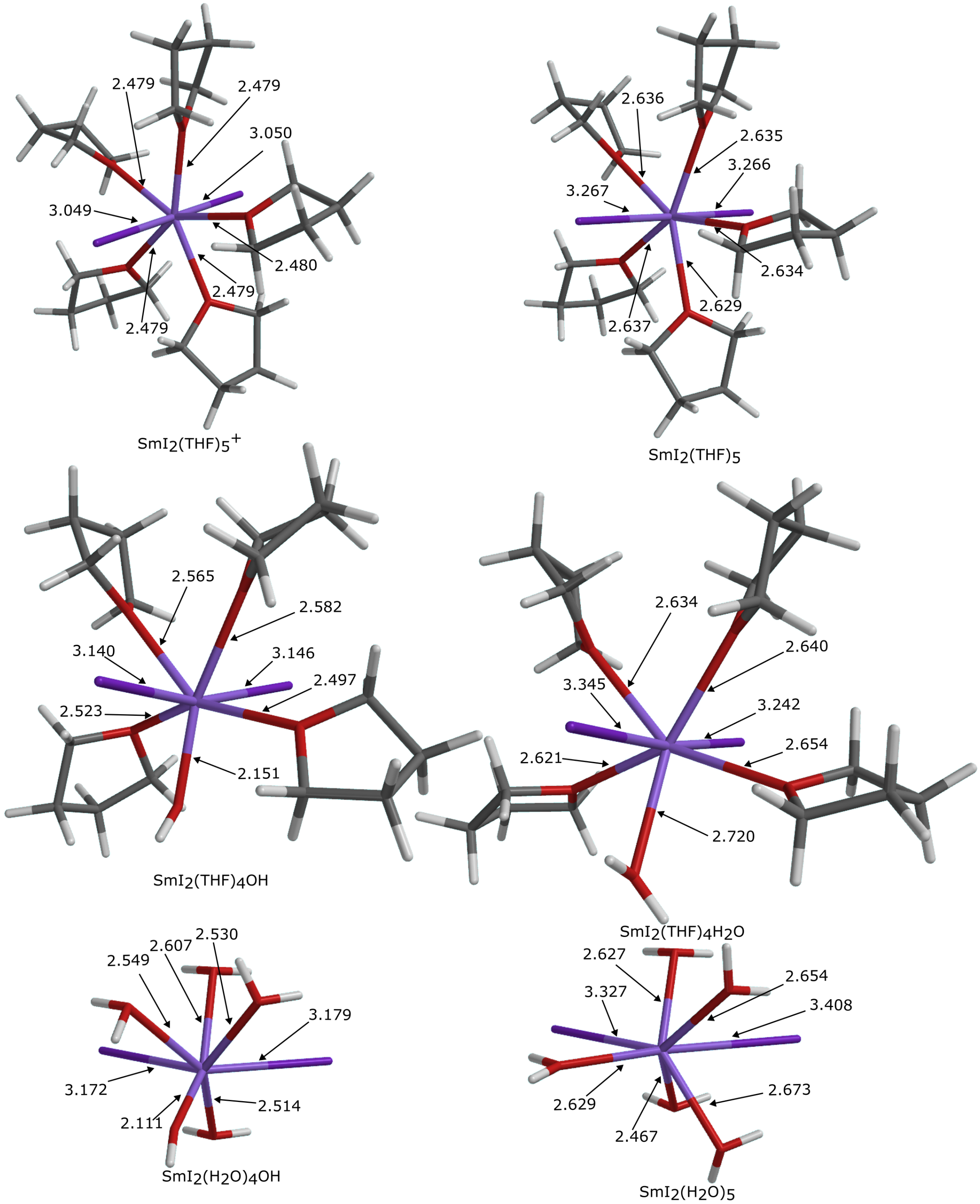

Figure S1. 3D geometries of the Sm complexes, with Sm–ligand bond distances (Å) indicated using arrows. Color code: oxygen, red; iodine dark purple; samarium light purple; carbon, grey; hydrogen, white.

In order to evaluate the quality of the geometry-optimized structures, the optimized geometry of $SmI_2(THF)_5^+$ was compared with the single-crystal X-ray diffraction structure reported for $[SmI_2(THF)_5][Co(CO)_4]$.[41] The computed Sm–I bond distances (3.050 Å and 3.049 Å) are only slightly longer than their experimental counterparts (3.030 Å and 3.009 Å).

Similarly, the calculated Sm–O bond distances (average: 2.479 Å) are slightly longer than their experimental counterparts (average: 2.456 Å). Also, the angles compare very well with the calculated I-Sm-I bond angle of 179.9° being within one degree of the crystal structure (178.9°). Furthermore, the mixture of SmI_2 and one equivalent of water has recently been studied using in situ extended X-ray absorption fine structure (EXAFS) spectroscopy.[42] Here, it was found that the average Sm-I distance was 3.25 Å, which is slightly shorter than the computed average distance of 3.294 Å of the $SmI_2(THF)_4H_2O$ complex. The measured average Sm–O distance of 2.63 Å is also only slightly shorter than the computed average distance of 2.654 Å.

In conclusion, the computational protocol used for geometry optimization, including large-core-ECPs for Sm, offers a geometry of $SmI_2(THF)_5^+$ that compares very well with that obtained from single-crystal X-ray diffraction. Furthermore, the computed average Sm-I and Sm-O bond lengths of $SmI_2(THF)_4H_2O$ compare extremely well with in situ EXAFS spectroscopy on the mixture of SmI_2 and one equivalent of water in THF.

S2.2: REACTION POTENTIALS AND FREE ENERGIES

Table S4: Calculated Energies, Reaction Free Energies, and Reduction Potentials of $SmI_2(THF)_5$ in THF Continuum.

Method	E^{THF} ($SmI_2(THF)_5$) [au]	E^{THF} ($SmI_2(THF)_5^+$) [au]	ΔG [kcal/mol][a]	E^0 *vs.* Fc/Fc+ [V]
HF	-2401.059488	-2400.971805	-48.3	-2.83
LSDA	-2406.637070	-2406.479764	-92.0	-0.96
PBE	-2410.940208	-2410.782462	-92.3	-0.94
PBE-D3(BJ)	-2411.033238	-2410.883014	-87.5	-1.15
M06L-D3	-2412.719023	-2412.575962	-83.0	-1.34
TPPS-D3(BJ)	-2412.335080	-2412.192772	-82.6	-1.36
PW6B95-D3(BJ)	-2414.647568	-2414.493513	-89.9	-1.04
B3LYP-D3(BJ)	-2412.888790	-2412.739852	-86.7	-1.18
M06-D3	-2411.825176	-2411.648253	-104.3	-0.43
M062X-D3	-2411.822049	-2411.652323	-99.8	-0.62
M06HF-D3	-2411.969313	-2411.827963	-82.0	-1.39
BHandHLYP	-2411.406384	-2411.269608	-79.1	-1.51
CAM-B3LYP	-2411.854815	-2411.696130	-92.8	-0.92
ωB97X-D	-2412.404350	-2412.250270	-90.0	-1.04
LC-ωHPBE	-2411.869381	-2411.693162	-103.8	-0.45
B2PLYP-D3(BJ)	-2410.557230	-2410.421228	-78.6	-1.53
PBEQIDH-D3(BJ)	-2410.000848	-2409.865671	-78.1	-1.55
DSD-PBEP86	-2176.473560	-2176.338267	-78.2	-1.55
revDSD-PBEP86	-2408.561192	-2408.429815	-75.7	-1.66

[a]Calculated using Equation 2 by including thermochemical corrections of $G^{THF\ 298K}_{PW6B95-D3(BJ)\ qh} = 0.529518$ au for $SmI_2(THF)_5$ and $G^{THF\ 298K}_{PW6B95-D3(BJ)\ qh} = 0.524516$ for $SmI_2(THF)_5^+$.

Table S5: Calculated Energies and BDFEs of $SmI_2(THF)_4H_2O$ in THF Continuum.

Method	E^{THF} ($SmI_2(THF)_4H_2O$) [au]	E^{THF} ($SmI_2(THF)_4OH$) [au]	E^{THF}($H^\bullet$) [au]	BDFE [kcal/mol][a]
HF	-2246.064167	-2245.510415	-0.499345	21.5
LSDA	-2251.432704	-2250.826394	-0.495756	56.7
PBE	-2255.086044	-2254.467520	-0.499214	62.2

PBE-D3(BJ)	-2255.164738	-2254.554769	-0.499214	56.8
M06L-D3	-2256.663990	-2256.061788	-0.502935	49.6
TPSS-D3(BJ)	-2256.211277	-2255.606655	-0.499424	53.4
PW6B95-D3(BJ)	-2258.366050	-2257.749667	-0.500878	59.8
B3LYP-D3(BJ)	-2256.791061	-2256.179683	-0.501740	56.1
M06HF-D3	-2255.914795	-2255.317565	-0.495810	51.0
BHandLYP	-2255.433414	-2254.837177	-0.498110	48.9
CAM-B3LYP	-2255.873507	-2255.258907	-0.498411	60.2
ωB97X-D	-2256.369791	-2255.752929	-0.502244	59.3
LC-ωHPBE	-2255.895667	-2255.261023	-0.505584	68.3
B2PLYP-D3(BJ)	-2253.858130	-2253.267429	-0.498177	45.4
PBEQIDH-D3(BJ)	-2253.212892	-2252.608432	-0.501319	36.2
DSD-PBEP86	-2252.876633	-2252.298450	-0.498535	37.3
revDSD-PBEP86	-2252.793460	-2252.221123	-0.498508	33.7

[a]Calculated using Equation 2 by including thermochemical corrections of $G^{\mathrm{THF\ 298K}}_{\mathrm{PW6B95-D3(BJ)\ qh}} = 0.438729$ au for $SmI_2(THF)_4H_2O$, $G^{\mathrm{THF\ 298K}}_{\mathrm{PW6B95-D3(BJ)\ qh}} = 0.431916$ au for $SmI_2(THF)_4OH$, and $G^{\mathrm{THF\ 298K}}_{\mathrm{PW6B95-D3(BJ)\ qh}} = -0.010654$ au for $H^{\bullet}$.

Table S6: Calculated Gas-Phase Energies and BDFEs of $\mathbf{SmI_2(H_2O)_5}$.

Method	E^{GAS} ($SmI_2(H_2O)_5$) [au]	E^{GAS} ($SmI_2(H_2O)_4OH$) [au]	E^{GAS}($H^{\bullet}$)[au]	BDFE [kcal/mol][a]
M06L-D3	-1632.420253	-1631.826235	-0.503425	41.7
PW6B95-D3(BJ)	-1633.192612	-1632.589619	-0.501301	48.7
M06HF-D3	-1631.668050	-1631.076151	-0.496173	44.9
B2PLYP-D3(BJ)	-1630.844157	-1630.261135	-0.498614	37.8
PBEQIDH-D3(BJ)	-1630.630444	-1630.047585	-0.501772	35.7
DSD-PBEP86	-1629.763824	-1629.194826	-0.498978	28.8
revDSD-PBEP86	-1629.692371	-1629.130095	-0.498951	24.6
CCSD	-1628.370622	-1627.800232	-0.499810	29.1
CCSD(T)	-1628.457861	-1627.888745	-0.499810	28.3

[a]Calculated using Equation 4 by including thermochemical corrections of $G^{\mathrm{THF\ 298K}}_{\mathrm{PW6B95-D3(BJ)\ qh}} = 0.073679$ au for $SmI_2(H_2O)_5$, $G^{\mathrm{THF\ 298K}}_{\mathrm{PW6B95-D3(BJ)\ qh}} = 0.065892$ au for $SmI_2(H_2O)_4OH$, and $G^{\mathrm{THF\ 298K}}_{\mathrm{PW6B95-D3(BJ)\ qh}} = -0.010654$ au for $H^{\bullet}$.

S2.3: DETERMINATION OF BASIS SET SUPERPOSITION ERRORS

Table S7: Calculated Electronic Energies of $\mathbf{SmI_2(H_2O)_5}$ and the Resulting BSSE.

Method	E^{GAS} ($SmI_2(H_2O)_4OH$) + ghost1 [au]	E^{GAS}($H^{\bullet}$) + ghost2 [au]	E^{GAS} ($SmI_2(H_2O)_4OH$) [au]	E^{GAS}($H^{\bullet}$) [au]	BSSE [kcal/mol]
M06L-D3	-1631.773683	-0.503733	-1631.773566	-0.503425	0.3
PW6B95-D3(BJ)	-1632.511904	-0.501418	-1632.511800	-0.501301	0.1
DSD-PBEP86	-1629.104898	-0.499015	-1629.103856	-0.498978	0.7

Table S8: Calculated Electronic Energies of $SmI_2(THF)_4H_2O$ and the Resulting BSSE.

Method	E^{GAS} ($SmI_2(THF)_4OH$) + ghost1 [au]	$E^{GAS}(H^{\bullet})$ + ghost2 [au]	E^{GAS} ($SmI_2(THF)_4OH$) [au]	$E^{GAS}(H^{\bullet})$ [au]	BSSE [kcal/mol]
M06L-D3	-2255.978144	-0.503767	-2255.977940	-0.503425	0.3
PW6B95-D3(BJ)	-2257.651104	-0.501424	-2257.650920	-0.501301	0.2
DSD-PBEP86	-2252.186374	-0.499018	-2252.185305	-0.498978	0.7

S2.4: DETERMINATION OF THE INFLUENCE OF SPIN ORBIT COUPLING

Table S9: Calculation of the SOC-Induced Stabilization of the Sm(II) Ground State.

System	CASSCF(6,7) averaged over 7 roots [au]	NEVPT2, lowest lying root [au]	QDPT with NEVPT2 diagonal energies, lowest eigenvalue of SOC matrix [au]	Spin-orbit stabilization of ground state [cm^{-1}]
Sm^{2+} in gas phase	-10417.123360	-10418.284049	-10418.294232	2234.97
Sm^{2+} in THF	-10417.521445	-10418.684153	-10418.694324	2232.27
$SmI_2(H_2O)_5$ in THF	-25019.347614	-25022.387662	-25022.397004	2050.50

Table S10: Calculation of the SOC-Induced Stabilization of the Sm(III) Ground State.

System	CASSCF(5,7) averaged over 21 roots [au]	NEVPT2, lowest lying root [au]	QDPT with NEVPT2 diagonal energies, lowest eigenvalue of SOC matrix [au]	Spin-orbit stabilization of ground state [cm^{-1}]
Sm^{3+} in gas phase	-10416.318191	-10417.427332	-10417.444101	3680.29
Sm^{3+} in THF	-10417.216096	-10418.327234	-10418.344012	3682.18
$SmI_2(H_2O)_5{}^+$ in THF	-25019.244198	-25022.255493	-25022.271656	3547.44
$SmI_2(H_2O)_4OH$ in THF	-25018.79073	-25021.82028	-25021.836462	3550.52

S3: REFERENCES

(1) Dunning, T. H. Gaussian Basis Sets for Use in Correlated Molecular Calculations. I. The Atoms Boron through Neon and Hydrogen. *J. Chem. Phys.* **1989**, *90*, 1007–1023.
(2) Kendall, R. A.; Dunning, T. H.; Harrison, R. J. Electron Affinities of the First-Row Atoms Revisited. Systematic Basis Sets and Wave Functions. *J Chem Phys* **1992**, *96*, 6796–6806.
(3) Peterson, K. A.; Dunning, T. H. Accurate Correlation Consistent Basis Sets for Molecular Core–Valence Correlation Effects: The Second Row Atoms Al–Ar, and the First Row Atoms B–Ne Revisited. *J. Chem. Phys.* **2002**, *117*, 10548–10560.
(4) Woon, D. E.; Dunning, T. H. Gaussian Basis Sets for Use in Correlated Molecular Calculations. III. The Atoms Aluminum through Argon. *J. Chem. Phys.* **1993**, *98*, 1358–1371.
(5) Woon, D. E.; Dunning, T. H. Gaussian Basis Sets for Use in Correlated Molecular Calculations. V. Core-valence Basis Sets for Boron through Neon. *J. Chem. Phys.* **1995**, *103*, 4572–4585.
(6) Peterson, K. A.; Figgen, D.; Goll, E.; Stoll, H.; Dolg, M. Systematically Convergent Basis Sets with Relativistic Pseudopotentials. II. Small-Core Pseudopotentials and Correlation Consistent Basis Sets for the Post-d Group 16–18 Elements. *J. Chem. Phys.* **2003**, *119*, 11113–11123.
(7) Peterson, K. A.; Shepler, B. C.; Figgen, D.; Stoll, H. On the Spectroscopic and Thermochemical Properties of ClO, BrO, IO, and Their Anions. *J. Phys. Chem. A* **2006**, *110*, 13877–13883.
(8) Dolg, M.; Stoll, H.; Savin, A.; Preuss, H. Energy-Adjusted Pseudopotentials for the Rare Earth Elements. *Theor. Chim. Acta* **1989**, *75*, 173–194.
(9) Dolg, M.; Stoll, H.; Preuss, H. A Combination of Quasirelativistic Pseudopotential and Ligand Field Calculations for Lanthanoid Compounds. *Theor. Chim. Acta* **1993**, *85*, 441–450.
(10) Roothaan, C. C. J. New Developments in Molecular Orbital Theory. *Rev. Mod. Phys.* **1951**, *23*, 69–89.
(11) Vosko, S. H.; Wilk, L.; Nusair, M. Accurate Spin-Dependent Electron Liquid Correlation Energies for Local Spin Density Calculations: A Critical Analysis. *Can J Phys* **1980**, *58*, 1200–1211.
(12) Hohenberg, P.; Kohn, W. Inhomogeneous Electron Gas. *Phys. Rev.* **1964**, *136*, B864–B871.
(13) Kohn, W.; Sham, L. J. Self-Consistent Equations Including Exchange and Correlation Effects. *Phys. Rev.* **1965**, *140*, A1133–A1138.
(14) Perdew, J.; Burke, K.; Ernzerhof, M. Generalized Gradient Approximation Made Simple. *Phys. Rev. Lett.* **1996**, *77*, 3865–3868.
(15) Perdew, J.; Burke, K.; Ernzerhof, M. Generalized Gradient Approximation Made Simple [Phys. Rev. Lett. 77, 3865 (1996)]. *Phys. Rev. Lett.* **1997**, *78*, 1396.
(16) Zhao, Y.; Truhlar, D. G. A New Local Density Functional for Main-Group Thermochemistry, Transition Metal Bonding, Thermochemical Kinetics, and Noncovalent Interactions. *J. Chem. Phys.* **2006**, *125*, 194101-1–18.
(17) Tao, J. M.; Perdew, J. P.; Staroverov, V. N.; Scuseria, G. E. Climbing the Density Functional Ladder: Nonempirical Meta-Generalized Gradient Approximation Designed for Molecules and Solids. *Phys. Rev. Lett.* **2003**, *91*.
(18) Zhao, Y.; Truhlar, D. G. Design of Density Functionals That Are Broadly Accurate for Thermochemistry, Thermochemical Kinetics, and Nonbonded Interactions. *J. Phys. Chem. A* **2005**, *109*, 5656–5667.
(19) Becke, A. D. Density-functional Thermochemistry. III. The Role of Exact Exchange. *J. Chem. Phys.* **1993**, *98*, 5648–5652.
(20) Zhao, Y.; Truhlar, D. G. The M06 Suite of Density Functionals for Main Group Thermochemistry, Thermochemical Kinetics, Noncovalent Interactions, Excited States, and Transition Elements: Two New Functionals and Systematic Testing of Four M06-Class Functionals and 12 Other Functionals. *Theor. Chem. Acc.* **2008**, *120*, 215–241.
(21) Zhao, Y.; Truhlar, D. G. Comparative DFT Study of van Der Waals Complexes: Rare-Gas Dimers, Alkaline-Earth Dimers, Zinc Dimer, and Zinc-Rare-Gas Dimers. *J. Phys. Chem. A* **2006**, *110*, 5121–5129.
(22) Zhao, Y.; Truhlar, D. G. Density Functional for Spectroscopy: No Long-Range Self-Interaction Error, Good Performance for Rydberg and Charge-Transfer States, and Better Performance on Average than B3LYP for Ground States. *J. Phys. Chem. A* **2006**, *110*, 13126–13130.
(23) Becke, A. D. A New Mixing of Hartree–Fock and Local Density-functional Theories. *J. Chem. Phys.* **1993**, *98*, 1372–1377.
(24) Yanai, T.; Tew, D. P.; Handy, N. C. A New Hybrid Exchange–Correlation Functional Using the Coulomb-Attenuating Method (CAM-B3LYP). *Chem. Phys. Lett.* **2004**, *393*, 51–57.
(25) Chai, J.-D.; Head-Gordon, M. Long-Range Corrected Hybrid Density Functionals with Damped Atom–Atom Dispersion Corrections. *Phys. Chem. Chem. Phys.* **2008**, *10*, 6615–6620.
(26) Henderson, T. M.; Izmaylov, A. F.; Scalmani, G.; Scuseria, G. E. Can Short-Range Hybrids Describe Long-Range-Dependent Properties? *J. Chem. Phys.* **2009**, *131*, 044108.
(27) Goerigk, L.; Grimme, S. Efficient and Accurate Double-Hybrid-Meta-GGA Density Functionals—Evaluation with the Extended GMTKN30 Database for General Main Group Thermochemistry, Kinetics, and Noncovalent Interactions. *J. Chem. Theory Comput.* **2011**, *7*, 291–309.
(28) Kozuch, S.; Martin, J. M. L. DSD-PBEP86: In Search of the Best Double-Hybrid DFT with Spin-Component Scaled MP2 and Dispersion Corrections. *Phys. Chem. Chem. Phys.* **2011**, *13*, 20104–20107.
(29) Santra, G.; Sylvetsky, N.; Martin, J. M. L. Minimally Empirical Double-Hybrid Functionals Trained against the GMTKN55 Database: RevDSD-PBEP86-D4, RevDOD-PBE-D4, and DOD-SCAN-D4. *J. Phys. Chem. A* **2019**, *123*, 5129–5143.
(30) Brémond, É.; Sancho-García, J. C.; Pérez-Jiménez, Á. J.; Adamo, C. Communication: Double-Hybrid Functionals from Adiabatic-Connection: The QIDH Model. *J. Chem. Phys.* **2014**, *141*, 031101.
(31) Grimme, S.; Antony, J.; Ehrlich, S.; Krieg, H. A Consistent and Accurate Ab Initio Parametrization of Density Functional Dispersion Correction (DFT-D) for the 94 Elements H-Pu. *J. Chem. Phys.* **2010**, *132*, 154104.

(32) Johnson, E. R.; Becke, A. D. A Unified Density-Functional Treatment of Dynamical, Nondynamical, and Dispersion Correlations. II. Thermochemical and Kinetic Benchmarks. *J Chem Phys* **2008**, *128*, 124105.
(33) Grimme, S.; Ehrlich, S.; Goerigk, L. Effect of the Damping Function in Dispersion Corrected Density Functional Theory. *J. Comput. Chem.* **2011**, *32*, 1456–1465.
(34) Sancho-García, J. C.; Brémond, É.; Savarese, M.; Pérez-Jiménez, A. J.; Adamo, C. Partnering Dispersion Corrections with Modern Parameter-Free Double-Hybrid Density Functionals. *Phys. Chem. Chem. Phys.* **2017**, *19*, 13481–13487.
(35) Purvis, G. D.; Bartlett, R. J. A Full Coupled-cluster Singles and Doubles Model: The Inclusion of Disconnected Triples. *J. Chem. Phys.* **1982**, *76*, 1910–1918.
(36) Scuseria, G. E.; Janssen, C. L.; Schaefer, H. F. An Efficient Reformulation of the Closed-shell Coupled Cluster Single and Double Excitation (CCSD) Equations. *J. Chem. Phys.* **1988**, *89*, 7382–7387.
(37) Scuseria, G. E.; Schaefer, H. F. Is Coupled Cluster Singles and Doubles (CCSD) More Computationally Intensive than Quadratic Configuration Interaction (QCISD)? *J. Chem. Phys.* **1989**, *90*, 3700–3703.
(38) Pople, J. A.; Head-Gordon, M.; Raghavachari, K. Quadratic Configuration Interaction. A General Technique for Determining Electron Correlation Energies. *J. Chem. Phys.* **1987**, *87*, 5968–5975.
(39) Cao, X. Y.; Dolg, M. Segmented Contraction Scheme for Small-Core Lanthanide Pseudopotential Basis Sets. *J. Mol. Struct.-Theochem* **2002**, *581*, 139–147.
(40) Dolg, M.; Stoll, H.; Preuss, H. ENERGY-ADJUSTED ABINITIO PSEUDOPOTENTIALS FOR THE RARE-EARTH ELEMENTS. *J. Chem. Phys.* **1989**, *90*, 1730–1734.
(41) Evans, W. J.; Bloom, I.; Grate, J. W.; Hughes, L. A.; Hunter, W. E.; Atwood, J. L. Synthesis and Characterization of the Samarium-Cobalt Complexes $(C_5Me_5)_2$(THF) $SmCo(CO)_4$ and $[SmI_2(THF)_5][Co(CO)_4]$: X-Ray Crystal Structure of a Seven-Coordinate Samarium(III) Cation Complex. *Inorg. Chem.* **1985**, *24*, 4620–4623.
(42) Yamamoto, A.; Liu, X.; Arashiba, K.; Konomi, A.; Tanaka, H.; Yoshizawa, K.; Nishibayashi, Y.; Yoshida, H. Coordination Structure of Samarium Diiodide in a Tetrahydrofuran–Water Mixture. *Inorg. Chem.* **2023**.
